# Dataset for landscape pattern analysis from a climatic perspective

**DOI:** 10.1016/j.dib.2019.104187

**Published:** 2019-06-25

**Authors:** Szilárd Szabó, Balázs Deák, Zoltán Kovács, Ádám Kertész, Boglárka Bertalan-Balázs

**Affiliations:** a Department of Physical Geography and Geoinformatics, Faculty of Science and Technology, University of Debrecen, Egyetem tér 1, H-4032, Debrecen, Hungary; b MTA-DE Biodiversity and Ecosystem Services Research Group, Egyetem tér 1, H-4032, Debrecen, Hungary; c University of Debrecen, Doctoral School of Earth Sciences, Hungary; d Geographical Institute, Research Centre for Astronomy and Earth Sciences of the Hungarian Academy of Sciences, Budaörsi str. 45., H-1112, Budapest, Hungary

**Keywords:** NDVI, Trend, Climatic factors, R-squared, Pattern

## Abstract

Revealing the driving forces of changes in landscape pattern is a key question of landscape ecology and landscape analysis. Temperature and precipitation as climatic variables have a dominant role in triggering vegetation changes; thus, a database, which contain their interaction, can support the understanding of spatio-temporal changes in vegetation patterns even on a large scale. The dataset provided in this article contain the R-squared values of bivariate linear regression analysis between the Normalized Difference Vegetation Index (target variable; as a general quantitative descriptor of surface greenness) of the TERRA satellite's MODIS sensor and the climatic variables of the CarpatClim database (predictor variables; maximum monthly temperature, aridification index, evapotranspiration and precipitation). Environmental variables are also included to support further analysis: terrain height, macro regions, land cover classes. The dataset has a spatial projection (i.e. maps) and covers the area of Hungary. Tabular version provides the possibility of traditional statistical analysis, while maps allow the investigation to involve the spatial characteristics of absolute and relative position of the data points. This data article is related to the paper “NDVI dynamics as reflected in climatic variables: spatial and temporal trends – a case study of Hungary” (Szabo et al., 2019).

**Table:** Specifications table

Subject area	*Environmental science*
More specific subject area	*Climate and vegetation*
Type of data	*Table and ESRI shape file*
How data was acquired	*Calculated regression coefficients from tabular data of CarpatClim database and from satellite image product of MODIS MOD13Q1 250 m database*
Data format	*Calculated*
Experimental factors	*CarpatClim is a spatial climatic database covering the period from 1961 to 2010 and providing data in a 10 km resolution grid. Aridification index, precipitation, potential evapotranspiration and maximum temperature data provided by the CarpatClim were involved in the analysis on a monthly basis.* *We also involved MODIS satellite's MOD13Q1 16 days 250 m composite product, which provided normalized differenced vegetation index data (NDVI) from 2000. NDVI data was assigned with the 10 km grid of climatic variables.* *Macro regions of Hungary, CORINE Land Cover categories and topographic (surface height, slope gradient and slope aspect derived from the SRTM digital surface model) data were also added to the database for each point of the grid.*
Experimental features	*Bivariate linear regression analyses were performed at each 1038 point of the CarpatClim database using the climatic variables and the MODIS MOD13Q1 data. Accordingly, we used data from 2000 till 2010, as this period was covered by both dataset.*
Data source location	*Hungary; N: 48.449,21.144; S: 45.845,18.390; W: 47.050,15.986; E: 47.233,23.396;*
Data accessibility	*Data can be downloaded from a public repository* *Szabó, Szilárd; Balázs, Boglárka; Kovács, Zoltán; Deák, Balázs; Kertész, Ádám (2019), “Dataset for landscape pattern analysis: relationships of climatic variables and NDVI ″, Mendeley Data, V1**https://doi.org/10.17632/3d6gcmx55y.1*
Related research article	*Data presented in this brief is related to the study:**Szabó, Szilárd*; *László, Elemér*; *Kovács, Zoltán*; *Püspöki, Zoltán*; *Kertész, Ádám*; *Singh, Sudhir Kumar;* *Balázs, Boglárka**(2019)* *NDVI dynamics as reflected in climatic variables: spatial and temporal trends – a case study of Hungary* *GISCIENCE AND REMOTE SENSING 56 : 4 pp. 624–644.**https://doi.org/10.1080/15481603.2018.1560686*

**Table:** 

**Value of the data** 1. It is a unique database of the relationship between the Normalized Difference Vegetation Index (NDVI) and the climatic variables especially relevant for the vegetation (aridification index – ARI, precipitation – PREC, potential evapotranspiration – PET and maximum temperature – TMAX). 2. Visual representation of the data by the maps we provided supports the evaluation of spatial patterns. 3. Data is available for researchers in its present form for further analysis and can also be extended with new variables using the spatial information. 4. R^2^ values support ecological studies focusing on vegetation dynamics as they can directly be used as explanatory variables. They can also be used as proxies for studies concerning future scenarios of climate change. 5. The methodology used in this paper provides a potential template for similar projects even outside the region; besides, the data might be valuable for meta-analyses.

## 1 Data

Landscape change and landscape pattern analysis is a key issue of landscape ecological research. Usually, landscape change can consider only a few dates with maps of field surveys or classified satellite images. Pattern is also important, but we often lack the appropriate data to analyse, and the classified maps also contain thematic errors [1], [2]. Using a time series of vegetation and climatic data, determining their relationships and to visualize the result on maps is a new approach to provide a solution to demonstrate spatially the climatic effects on the vegetation. This approach enables to study the spatial pattern and to involve the strength of correlation into a deeper analysis with topographic, biogeographic or even social factors.

**Fig. 1:**
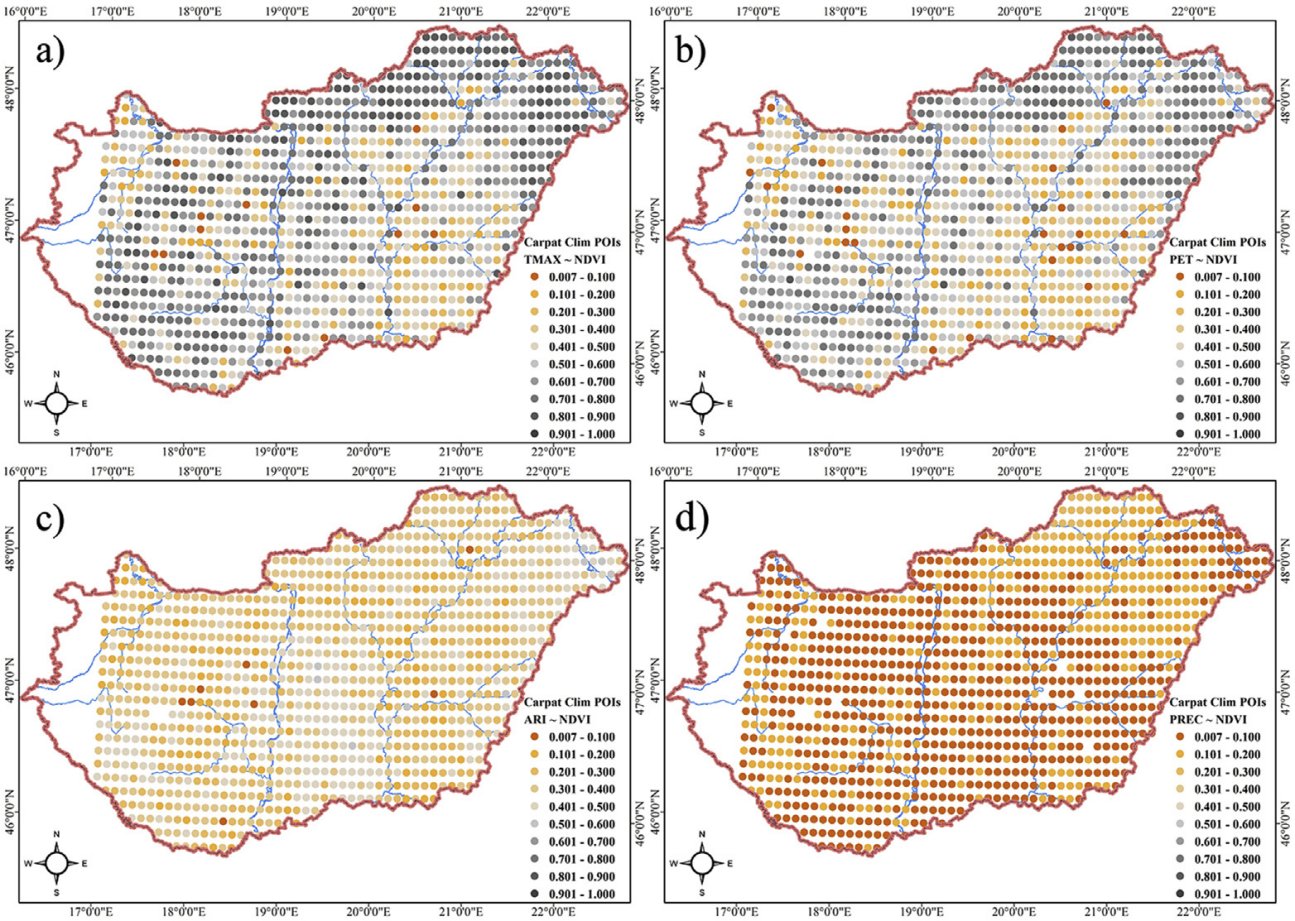
R-squared values of the bivariate regression analyses between a: NDVI and monthly temperature maximum, b: NDVI and potential evapotranspiration; c: NDVI and aridification index; d: NDVI and precipitation.

Our database reflects the relationship of climatic variables with the vegetation (NDVI); i.e. how the vegetation cover is determined by the climatic factors (Fig. 1). Supplementary environmental factors help to reveal the patterns of this relationship (Table S1). It can also be useful for the exploration of the regions where NDVI has a high dependence on aridity, evapotranspiration, precipitation or monthly maximum temperature. As it is a spatial dataset, the relationships can also be studied with measures of spatial indices such as autocorrelation or pattern analysis (e.g. is the data isotropic or anisotropic; is there a stationarity) [3].

## 2 Experimental design, materials, and methods

Climatic data was represented by the CarpatClim (CC) database of nine Central-European countries [4], [5]. It is a database containing several climatic variables in daily, monthly or yearly basis from 1960 to 2010. All data is based on measurements of meteorological stations and presented in a regular grid of 10 km × 10 km. Data, at first, were homogenized using the Multiple Analysis of Series for Homogenization method (MASH) [6], [7], [8] and then were interpolated by the Meteorological Interpolation based on Surface Homogenized Data Basis (MISH) [9]. Homogenization ensured the harmonization of the different measurement technologies of the countries involved in the program and managed the missing data completion. The interpolation technique was developed directly for climatic data using long-term observations (i.e. time series) beside the spatial parameter [9]. The application of the MASH and MISH approaches resulted in a reliable spatial prediction: representativeness of the data was between (70–85%) [7].

Normalized Difference Vegetation Index (NDVI) data were provided by the MOD13Q1 16-days product with 250 m spatial resolution [10]. NDVI reflects the greenness of the surface, i.e. has a direct relationship with the vegetation cover [11]. Its values are calculated using the formula in Eq. (1).

(1) $$NDVI=\;\frac{infra\;red-red}{infra\;red+red}$$

Where infrared: is the infrared range of the electromagnetic spectrum (620–670 nm in case of MODIS sensor); red: is the red spectrum of the electromagnetic spectrum (841–876 nm in case of MODIS sensor).

As NDVI is a normalized index, it ranges between −1 and +1. We extracted the data to fit into the 10 × 10 km grid of the CC.

As supplementary data, we also involved topographic data into the dataset using the SRTM digital surface model [12] with terrain height, slope and aspect. Besides, nominal data, as factors for further analysis, were added: (1) land cover data from CORINE Land Cover [13] database with the CLC codes and with aggregated categories (arable lands, grasslands, forests, wetlands, water bodies and artificial surfaces); and (2) macro regions of Hungary (Great Hungarian Plain, Kisalföld, Transdanubian Mountains, North-Hungarian Mountains, Alpokalja, Transdanubian Hills; according to Dövényi [14]). All supplementary data were extracted for the 10 × 10 km grid of the CC.

Bivariate linear regression analyses were performed for the CC-variables (fix factors) and the NDVI scores (dependent variable) in each CC-grid (1038 points). Influential data, which can distort the results, were filtered out based on the Cook's distance (D), excluding cases where D > 4/n (n: number of cases) [15]. We reported the R^2^-values for each point of the CC-grid (Fig. 1).

Statistical analysis was performed in R 3.4 [16].
